# Deficient Adipogenesis of Scleroderma Patient and Healthy African American Monocytes

**DOI:** 10.3389/fphar.2017.00174

**Published:** 2017-04-03

**Authors:** Rebecca Lee, Charles Reese, Gustavo Carmen-Lopez, Beth Perry, Michael Bonner, Marina Zemskova, Carole L. Wilson, Kristi L. Helke, Richard M. Silver, Stanley Hoffman, Elena Tourkina

**Affiliations:** ^1^Division of Rheumatology and Immunology, Department of Medicine, Medical University of South CarolinaCharleston, SC, USA; ^2^Division of Pulmonary, Critical Care, Allergy and Sleep Medicine, Medical University of South CarolinaCharleston, SC, USA; ^3^Department of Comparative Medicine, Medical University of South CarolinaCharleston, SC, USA; ^4^Department of Regenerative Medicine and Cell Biology, Medical University of South CarolinaCharleston, SC, USA

**Keywords:** adipogenesis, fibrosis, caveolin-1, Peroxisome proliferator-activated receptor γ, monocytes

## Abstract

Monocytes from systemic sclerosis (SSc, scleroderma) patients and healthy African Americans (AA) are deficient in the regulatory protein caveolin-1 leading to enhanced migration toward chemokines and fibrogenic differentiation. While dermal fibrosis is the hallmark of SSc, loss of subcutaneous adipose tissue is a lesser-known feature. To better understand the etiology of SSc and the predisposition of AA to SSc, we studied the adipogenic potential of SSc and healthy AA monocytes. The ability of SSc and healthy AA monocytes to differentiate into adipocyte-like cells (ALC) is inhibited compared to healthy Caucasian (C) monocytes. We validated that monocyte-derived ALCs are distinct from macrophages by flow cytometry and immunocytochemistry. Like their enhanced fibrogenic differentiation, their inhibited adipogenic differentiation is reversed by the caveolin-1 scaffolding domain peptide (CSD, a surrogate for caveolin-1). The altered differentiation of SSc and healthy AA monocytes is additionally regulated by peroxisome proliferator-activated receptor γ (PPARγ) which is also present at reduced levels in these cells. *In vivo* studies further support the importance of caveolin-1 and PPARγ in fibrogenesis and adipogenesis. In SSc patients, healthy AA, and mice treated systemically with bleomycin, adipocytes lose caveolin-1 and PPARγ and the subcutaneous adipose layer is diminished. CSD treatment of these mice leads to a reappearance of the caveolin-1+/PPARγ+/FABP4+ subcutaneous adipose layer. Moreover, many of these adipocytes are CD45+, suggesting they are monocyte derived. Tracing experiments with injected EGFP+ monocytes confirm that monocytes contribute to the repair of the adipose layer when it is damaged by bleomycin treatment. Our observations strongly suggest that caveolin-1 and PPARγ work together to maintain a balance between the fibrogenic and adipogenic differentiation of monocytes, that this balance is altered in SSc and in healthy AA, and that monocytes make a major contribution to the repair of the adipose layer.

## Introduction

Scleroderma (systemic sclerosis, SSc) is a complex autoimmune disease involving fibrosis of the skin, lungs, and other organs. An underappreciated feature of SSc is loss of subcutaneous adipose tissue (Marangoni et al., 2015). This loss also occurs in mice when bleomycin is used to induce skin fibrosis (Lee et al., 2014a; Marangoni et al., 2015). As described below, two molecules of interest in the regulation of adipogenesis are caveolin-1 and peroxisome proliferator-activated receptor γ (PPARγ). Interestingly, both caveolin-1 and PPARγ are among the group of proteins in which mutations result in lipodystrophy (Rochford, 2010).

African Americans (AA) have an increased risk of developing SSc compared to Caucasians (C) (Tager and Tikly, 1999) as exemplified by a younger age of disease onset, higher probability of the more severe diffuse cutaneous disease, and higher mortality (Laing et al., 1997; Mayes, 2003; Krishnan and Furst, 2005; Nietert et al., 2006). While there has been a major focus on AA SSc patients, there have been few studies on underlying differences between healthy AA and C that might result in this predisposition. In one study, healthy AA were found to have twice the serum level of the profibrotic cytokine TGFβ than healthy C (August and Suthanthiran, 2003). We have identified several parameters in which healthy AA are similar to SSc patients (Silver et al., 2012; Reese et al., 2014b) which may contribute to the predisposition of AA to SSc.

Caveolin-1, a protein associated with plasma membrane invaginations known as caveolae and with other cellular membranes is underexpressed on various cell types from healthy AA and SSc patients including fibroblasts and monocytes (Tourkina et al., 2005, 2008, 2010; Del Galdo et al., 2008). Similarly, caveolin-1 is also deficient in mice in which skin and lung fibrosis have been induced with bleomycin (Kasper et al., 1998; Del Galdo et al., 2008; Tourkina et al., 2008; Lee et al., 2014a,c). The caveolin-1 scaffolding domain peptide (CSD) can enter cells and inhibit kinases just like full-length caveolin-1 (Bucci et al., 2000; Bernatchez et al., 2005). Through its ability to act as a surrogate for caveolin-1, CSD can reverse a variety of cell behaviors associated with fibrosis *in vitro* (collagen overexpression by SSc fibroblasts, enhanced migration and enhanced fibrocyte differentiation by AA and SSc monocytes (Tourkina et al., 2005, 2011; Reese et al., 2014b) and *in vivo* (enhanced lung and dermal fibrosis, loss of subcutaneous adipocytes, Lee et al., 2015).

PPARγ is a ligand-activated nuclear receptor that regulates diverse aspects of lipid and lipoprotein metabolism and glucose homeostasis (Jiang et al., 1998; Ricote et al., 1998; Evans et al., 2004; Berger et al., 2005). In addition to expression in adipocytes, PPARγ is expressed in endothelial cells, vascular smooth muscle cells, monocytes/macrophages, and fibroblasts (Collins et al., 2001; Ghosh et al., 2004). Endogenous and diet-derived fatty acids and eicosanoids such as prostaglandin J2 are low-affinity natural PPARγ ligands; the thiazolidenedione drugs [e.g., rosiglitazone and troglitazone (TRO)] are potent synthetic PPARγ agonists (Spiegelman, 1998).

Monocytes and monocyte-derived fibrocytes have been reported to differentiate into both adipocytes and myofibroblasts (Kuwana et al., 2003; Hong et al., 2005, 2007). Here we have studied the differentiation of monocytes into ALCs in fibrotic disease using both human samples (from healthy C, healthy AA, and SSc patients) and mice treated with bleomycin or vehicle. These studies indicate that healthy AA and SSc monocytes are deficient in adipogenic differentiation due to the low levels of caveolin-1 and PPARγ in these cells. Human studies further demonstrate that monocyte-derived ALCs are readily distinguished from macrophages. Mouse experiments demonstrate that monocytes contribute to the subcutaneous adipose cell layer, particularly the regeneration of this layer following bleomycin-induced injury. In summary, these studies support the concept that fibrotic skin disease results in part from the fibrogenic differentiation of monocytes at the expense of their adipogenic differentiation and that caveolin-1 and PPARγ are two key proteins in regulating this balance.

## Methods

### Blood donors

Under a protocol approved by the Medical University of South Carolina (MUSC) Institutional Review Board for Human Research, SSc interstitial lung disease (ILD) patients were recruited from the MUSC Scleroderma Clinic. All patients provided written informed consent before enrollment in the study and fulfilled the American College of Rheumatology criteria for SSc (Subcommittee, 1980) and had evidence of ILD (Tourkina et al., 2010). Demographic data for SSc patients and healthy control donors are summarized in Tables 1, 2. Note that Table 1 describes the combined data for all the patients that participated in the entire study, not the patients that participated in a particular experiment. Healthy C and AA are studied separately because we have previously published that there are major differences in their monocytes in caveolin-1 levels and cell function (Reese et al., 2014b). However, the data for C and AA SSc patients are not separated because no differences have been observed (Reese et al., 2014b).

**Table 1:** **Clinical features of SSc patients**.

**Race/Smoking**	**Gender**	**Numbers**
Caucasian	M	3
Caucasian	F	14
African American	M	0
African American	F	12
Smoker		1
Former Smoker		2
	**C**	**AA**
Age: Mean ± SD (range)	57.9 ± 9.5 (43–75)	49.7 ± 11.7 (33–72)
Disease duration: Mean ± SD (range)	9.4 ± 5.8 (5–22)	9.3 ± 8.7 (1–28)
Limited Cutaneous	11	7
Diffuse Cutaneous	6	5
Overlap	8	9
Pulmonary Involvement (ILD)	17/17 (100%)	12/12 (100%)

*Patients: SSc patients were classified as having either limited cutaneous (lcSSc) or diffuse cutaneous (dcSSc) disease according to (Leroy et al., 1988). Disease duration was determined based on when the first non-Raynaud's Phenomenon symptoms were documented. We present demographics on C and AA SSc patients separately here. However, because in our studies we found no differences between these groups, the data is pooled in the Results section*.

**Table 2:** **AA and caucasians controls**.

**Race**	**Gender**	**Donors**	**Smokers**	**Former smokers**
Caucasian	M	13	0	2
Caucasian	F	20	1	2
African American	M	4	0	1
African American	F	24	1	2

*Age: Mean ± SD (range); Caucasian 34.4 ± 10.9 (19-57); African American 36.6 ± 11.2 (21-57)*.

### PBMC and monocyte isolation

PBMC were isolated by standard methods (Tourkina et al., 2010) by centrifugation on density 1.083 Histopaque cushions. Monocytes were isolated from the PBMC by immunodepletion using a Dynal Monocyte Negative Isolation Kit (Invitrogen, Carlsbad, CA) resulting in a cell population about 95% Mac-1+ monocytes (Tourkina et al., 2010), then allowed to recover overnight in 6-well tissue culture plates (2 × 10^6^ cells per well) in RPMI 1640/ 20% FCS.

### Monocyte to ALC differentiation

Methods were slightly modified from Hong et al. (2007) and Kuwana et al. (2003). Briefly, PBMC were plated in six-well fibronectin-coated plates (2 × 10^7^ cells in 2 ml DMEM/ 20% FCS). On day 5, ALC differentiation was initiated by incubating the cells for 3 days in Adipocyte Induction Medium (AIM) described by Kuwana et al. (2003) (DMEM/ 10% FBS/ 1 μM TRO/ 1 μM Dexamethasone/ 100 μM Indomethacin/ 10 μg/ml Insulin) followed by 1 day in Maintenance Medium (DMEM/ 10% FBS/ 10 μg/ml Insulin). The cycle of “3 days on, 1 day off” was repeated and cells were harvested on day 13 cells. For CSD treatment, the medium was supplemented on days 2, 5, and 9 with a final 0.1 μM CSD (amino acids 82–101 of caveolin-1 (DGIWKASFTTFTVTKYWFYR-NH_2_) purchased from Elim Biopharmaceuticals). When treating cells with CSD, a stock solution (10 mM in 100% DMSO) was diluted to the indicated final concentration.

For staining, the same methods were used except that the wells contained fibronectin-coated coverslips. For Western blots, cells were extracted with the Lysis Buffer described below. Staining and Western blotting were performed as described below.

### Monocyte to macrophage differentiation

PBMC were plated in six-well tissue culture plates (1 × 10^7^ cells in 2 ml Monocyte Attachment Medium [Promocell]) for 1.5 h. The medium and unbound cells were removed and bound cells further incubated in Supplemented Basal Medium (Promocell) + M-CSF or GM-CSF (100 ng/ml). 1 ml fresh medium is added at day 6 (plus M-CSF or GM-CSF) and again at day 9. Cells are harvested on day 11 and analyzed by immunocytochemistry and flow cytometry (Reese et al., 2014a).

### Treatment of monocytes

After isolation and recovery as described above, cells were treated for 3 h in RPMI/ 1% BSA supplemented with 0.1 μM CSD or TRO (1 μM). In some experiments, cells were pretreated with TGFβ for 45 min. After incubation, cells were washed with cold PBS and extracted with Lysis Buffer (20 mM Tris-HCl (pH 7.5)/ 1% NP-40/ 100 mM NaCl/ 5 mM EDTA/ 2 mM KCl) supplemented with 1 mM phenylmethylsulfonyl fluoride, protease inhibitor mixture Set V (Calbiochem), and phosphatase inhibitors (10 mM sodium pyrophosphate, 5 mM NaF, 10 mM beta-glycerophosphate, and 10 mM sodium orthovanadate). For staining, the same methods were used except that the wells contained coverslips. Staining and Western blotting were performed as described below.

### Staining

Cells on coverslips were fixed with 4% paraformaldehyde, then stained with Oil Red O or blocked for 24 h with PBS/ 5% BSA/ 0.1% Triton X-100 then stained immunocytochemically using the indicated primary antibodies (Table 3) and appropriately conjugated secondary antibodies. Nuclei were counterstained using DAPI.

**Table 3A:** **Antibodies Used in Western Blotting**.

**Target**	**Source**	**Type**
Human PPARγ	Cell Signaling #2435	Rabbit monoclonal
Human Caveolin-1	Santa Cruz sc-894	Rabbit polyclonal
Human FABP4	EMD Millipore MABS172	Rabbit monoclonal
Human GAPDH	EMD Millipore MAB374	Mouse monoclonal

**Table 3B:** **Antibodies Used in Immunocytochemistry, Immunohistochemistry, and Flow Cytometry**.

**Target**	**Source**	**Type**
Mouse PPARγ	Abcam ab59256	Rabbit polyclonal
Mouse Caveolin-1	Santa Cruz sc-894	Rabbit polyclonal
Mouse Caveolin-1	BD Biosciences 610057	Mouse monoclonal
Mouse FABP4	Abcam ab93945	Mouse monoclonal
Mouse FABP4	EMD Millipore MABS172	Rabbit monoclonal
Mouse CD45	BD Biosciences 553076	Rat monoclonal
Human PPARγ	Abcam ab59256	Rabbit polyclonal
Human Caveolin-1	Santa Cruz sc-894	Rabbit polyclonal
Human Caveolin-1	BD Biosciences 610057	Mouse monoclonal
Human CD45	eBioscience 14-0459	Mouse monoclonal
Human FABP4	Abcam ab93945	Mouse monoclonal
Human CD14	Santa Cruz sc-9150	Rabbit polyclonal
Human CD163	Santa Cruz sc-20066	Mouse monoclonal
Human iNOS	BD Transduction Lab 610432	Mouse monoclonal
Human Arginase	eBioscience 14-9779-82	Mouse monoclonal
Human CD206	Santa Cruz sc-376232	Mouse monoclonal

### Western blotting

Protein concentrations in Lysis Buffer extracts were measured using the BCA™ Protein assay kit (Pierce). Up to 40 μg of total protein was loaded per lane. Western blotting was performed by routine methods using the indicated primary antibodies (Table 3).

### Mouse experiments

These studies were performed under protocols approved by the MUSC Institutional Animal Care & Use Committee. (1) Treatment of mice with bleomycin and CSD: Mice were treated systemically with bleomycin (Cipla, Mumbai, India) (67 U/kg) or vehicle and received CSD or vehicle as recently described (Lee et al., 2014a,b). Skin tissue sections were then stained with the indicated antibodies. (2) Injection of Monocytes Isolated from EGFP ± Mouse Bone Marrow: EGFP+ mice (Jackson Laboratory) were treated with bleomycin or vehicle as described above. On day 21, mice were sacrificed and monocytes were isolated from bone marrow: Femurs and tibias were dissected, scissors were used to gently snip off the ends of the bones, and BM cells were flushed out using MACS buffer (PBS [pH 7.2]/ 0.5% BSA/ 2 mM EDTA) delivered with a syringe and a 26G needle. The cells were disaggregated by gentle pipetting, then passed through a 40 μm cell strainer to remove residual cell clumps or debris. Cells were washed with MACS buffer and collected by centrifugation (300 × g, 10 min). Finally, monocytes were enriched by negative selection using a MACS Monocyte Isolation Kit (BM) mouse (130-100-629) following the manufacturer's protocol. The purity of the isolated monocytes was confirmed by flow cytometry. Of particular note, the cells were almost 100% CD45+, CD11b+, CD68+, CD73−, CD90−, and CD105−. Therefore, the population does not contain mesenchymal stromal cells (which are CD45−, CD73+, CD90+, and CD105+). Finally, cells (5 × 10^5^) in 200 μl PBS were injected into host mice (10 days after the initiation of bleomycin or vehicle treatment) via the retro-orbital sinus. Mice were sacrificed on day 28, skin tissue sections prepared, and analyzed by Masson's Trichrome Staining and fluorescence microscopy to detect EGFP+ cells and FABP4 by immunohistochemistry.

### Immunohistochemistry of mouse and human tissue sections

Immunohistochemistry of skin tissue sections was performed as described (Tourkina et al., 2011). Briefly, paraffin sections were stained using the indicated primary antibodies (Table 3), appropriate conjugated secondary antibodies, and the nuclear stain DAPI (Invitrogen, Carlsbad, CA).

### Statistical analyses

Immunoreactive bands were quantified by densitometry using Image J 1.32 NIH software. Raw densitometric data were processed and analyzed using Prism 3.0 (GraphPad Software Inc.) and normalized vs. data obtained with GAPDH as the loading control. *T*-test or one-way ANOVA with Tukey's correction *post-hoc* was used as appropriate to evaluate Western blots. *p* < 0.05 was considered as a statistically significant difference. In all figures, ^***^ indicates *p* < 0.001, ^**^ indicates *p* < 0.01, and ^*^ indicates *p* < 0.05.

## Results

We previously demonstrated that monocytes from SSc patients and healthy AA exhibit enhanced fibrogenic differentiation due to a deficiency in caveolin-1 that can be functionally reversed by treating the cells with CSD which serves as a surrogate for caveolin-1 (Reese et al., 2014b; Lee et al., 2015). Given that dermal fibrosis is associated with a loss of subcutaneous adipocytes both in SSc patients and in a mouse model for SSc (Lee et al., 2014a; Marangoni et al., 2015), here we have determined whether SSc and AA monocytes are also altered in their ability to differentiate into lipid-containing, FABP4+ cells which for simplicity we refer to as ALCs. Although other monocyte derivatives (macrophages) can accumulate lipid and express FABP4 (Tontonoz et al., 1998; Jiang et al., 2013; Lázaro et al., 2013; Dubland and Francis, 2015), we will show that ALCs derived from control monocytes contain much higher levels of FABP4 than do macrophages differentiated from monocytes from the same donor.

### Decreased ALC differentiation by SSc and healthy AA monocytes

Our method for studying monocyte differentiation into ALCs *in vitro* is modified from (Hong et al., 2007) and (Kuwana et al., 2003). ALC differentiation is evaluated in terms of histochemical staining with the lipid dye Oil Red O, immunocytochemical (ICC) staining for the adipocyte marker fatty acid binding protein 4 (FABP4), Western blotting, and flow cytometry.

**Figure 1:**
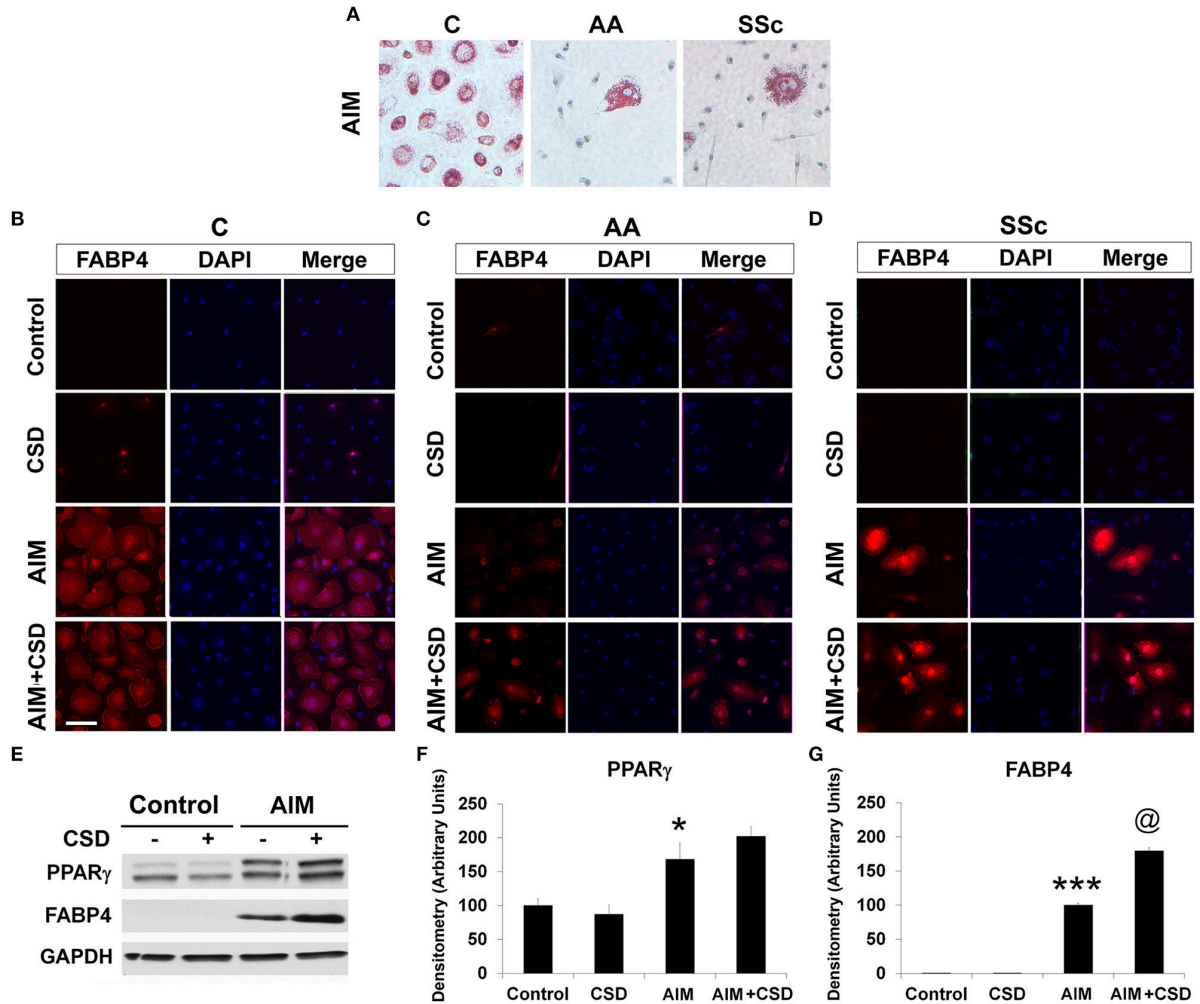
**Monocyte Differentiation into ALCs**. PBMC from healthy C, healthy AA, and SSc patients were incubated in medium that promote ALC differentiation (AIM) or in Control medium lacking additives. **(A)** Cultures were stained with Oil Red O to detect ALCs. Similar results were obtained in three independent experiments. **(B)** Healthy C, **(C)** healthy AA, and **(D)** SSc patient cultures were supplemented with CSD on days 2, 5, and 9. Cultures were stained for FABP4 to detect ALCs. Similar results were obtained in three independent experiments. **(E)** Lysis Buffer extracts of the indicated cultures from healthy C donors **(B)** were Western blotted with the indicated antibodies. GAPDH served as a loading control. **(F,G)** Western blots (*n* = 4 independent experiments using cells from different subjects) performed as in **(E)** were quantified densitometrically (average ± s.e.m.) normalized against GAPDH. In **(F)** the level of PPARγ in Control cultures was set to 100 Arbitrary Units. In **(G)** the level of FABP4 in AIM cultures was set to 100 Arbitrary Units. ^***^p < 0.001, ^*^p < 0.05 vs. Control; ^@^*p* < 0.05 vs. AIM.

Following treatment with Adipocyte Induction Medium (AIM), almost 100% of C cells are large and round with lipid droplets brightly stained with Oil Red O while such cells rare among AA and SSc cells (Figure 1A, Table 4). When ALC differentiation was detected by FABP4 staining (Figures 1B–D), no stained cells were observed under Control conditions (even among C cells). With induction of adipogenesis, the number of FABP4+ cells detected by ICC is much higher in C cells than among AA or SSc cells (Figures 1B–D, Table 4) indicating that, in accord with the Oil Red O staining, adipocyte differentiation is deficient among AA and SSc monocytes.

**Table 4:** **ALC Differentiation Detected by Oil Red O and FABP4 Staining**.

**PBMC**	**Oil Red O Stain**	**FABP4 Stain**			
	**Treatment**	**Treatment**			
	**AIM**	**Control**	**CSD**	**AIM**	**AIM + CSD**
C	30.0 ± 3.7	1.3 ± 0.5	2.3 ± 0.5	29 ± 6	40.0 ± 3
AA	4.4 ± 2.0^***^	0.6 ± 0.1	1.23 ± 0.05	7.6 ± 2.0^*^	21.3 ± 4^**^^,^^@@^
SSc	3.9 ± 2.8^***^	0 ± 0	1 ± 0.3	4.3 ± 0.3^*^	15.0 ± 1^**^^,^^^@@@^^

*The results of three independent experiments performed as in Figure 1A (for Oil Red O staining) and as in Figures 1B–D (for FABP4 staining) were quantified in terms of positive cells per field, six fields per subject*.

*** *p < 0.001*,

** *p < 0.01*,

* p < 0.05 for AA or SSc vs. C in each category;

@@@ *p < 0.001*,

@@ *p < 0.01 for AIM + CSD vs. AIM. Statistical analyses are not presented for the small number of positive cells under Control and CSD*.

**Figure 2:**
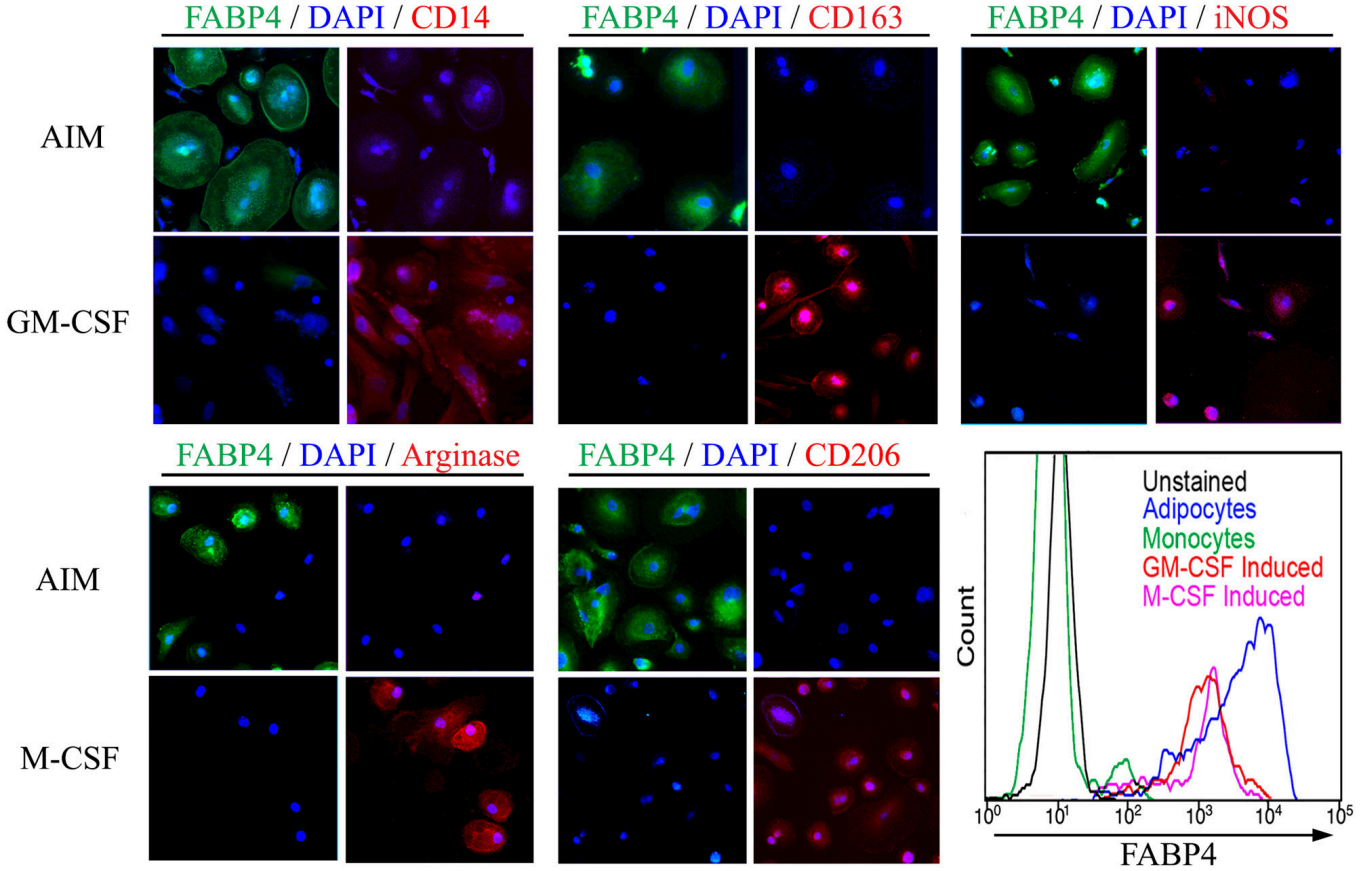
**Monocyte-Derived Macrophages and ALCs Carry Distinct Markers**. As described in the Methods, monocytes were induced to differentiate into ALCs using AIM or into macrophages using GM-CSF or M-CSF. Cells were then stained using the indicated antibodies and with DAPI. In each panel of four images, those on the left shows FABP4 + DAPI and those on the right show the indicated macrophage marker + DAPI. (Lower right) FABP4 levels in the indicated cell populations were determined by flow cytometry. Results are representative of three repeats in each category.

To distinguish adipocyte differentiation from macrophage differentiation, monocytes from the same donor were treated with AIM to induce adipocyte differentiation or with M-CSF or GM-CSF to induce macrophage differentiation. Despite their common origin, the markers expressed following these treatments are very different (Figure 2). ALCs are strongly FABP4+ while macrophages are CD163+, iNOS+, arginase+, CD206+ depending on whether they were induced with M-CSF or GM-CSF, and are routinely CD14+ (Kuwana et al., 2003). In contrast, ALCs are CD14−, CD163−, CD206−, arginase−, and iNOS−. Because it has been reported that macrophages (particularly foam cells) can be FABP4+ (Jiang et al., 2013; Lázaro et al., 2013), we quantified FABP4 levels by flow cytometry (Figure 2). While macrophages contain FABP4, its level in ALCs was much higher, indicating that FABP4 can be used as an ALC marker.

### Roles of caveolin-1 and PPARγ

Given that CSD, by acting as a surrogate for caveolin-1, reverses the enhanced ability of AA and SSc monocytes to differentiate into fibrocytes (Reese et al., 2014b), we hypothesized that CSD might also reverse the decreased ability of AIM-treated AA and SSc monocytes to differentiate into ALCs. Indeed, ICC (Figures 1D,E, Table 4) experiments demonstrated an increased number of large, round FABP4+ cells derived from AA and SSc monocytes in the presence of CSD. The ability of CSD to enhance ALC differentiation is also observed for C monocytes, both in ICC (Table 4) and Western blotting experiments for FABP4 (Figures 1E,G).

**Figure 3:**
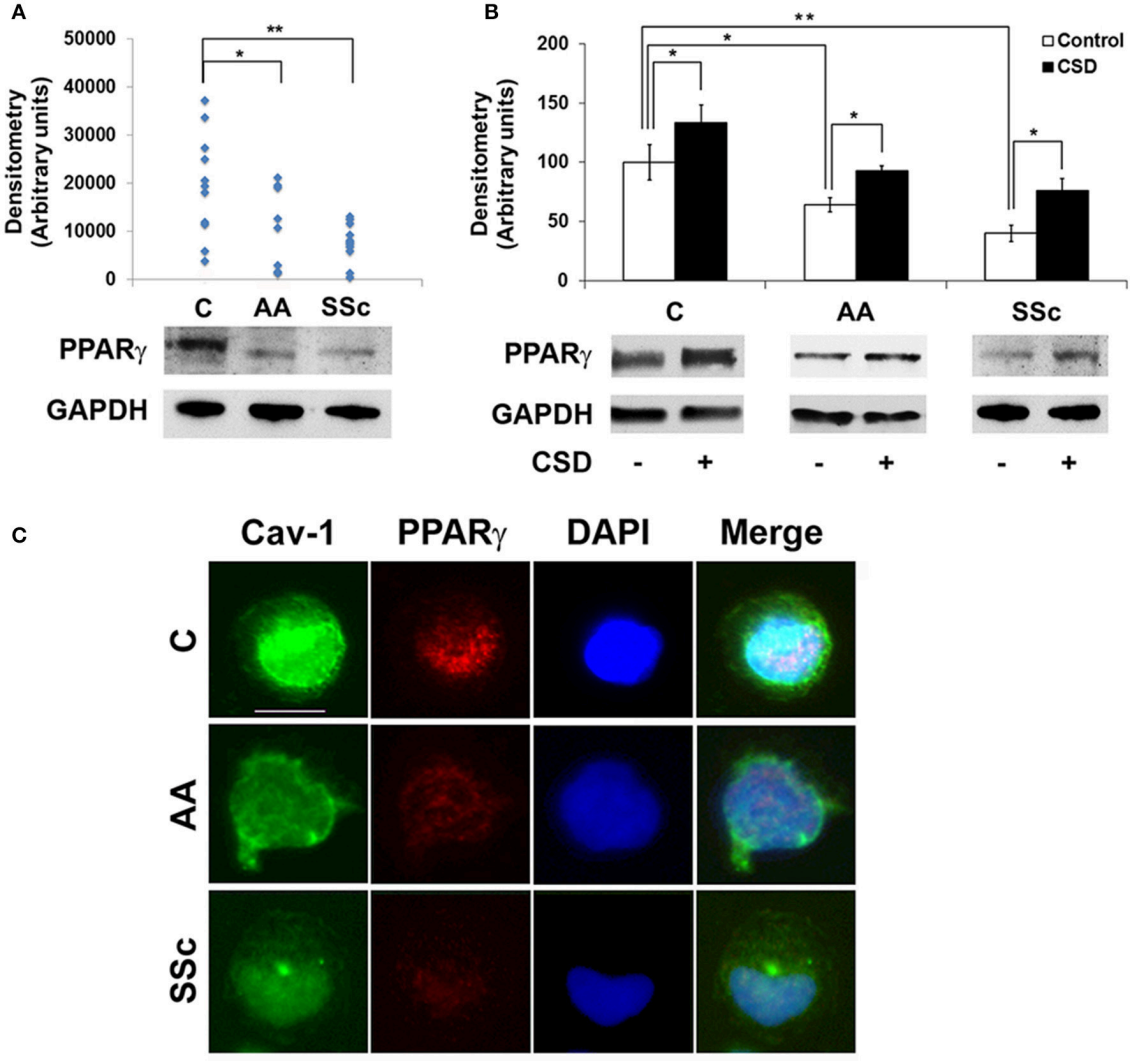
**Low Caveolin-1 and PPARγ in AA and SSc Monocytes. (A)** (bottom) Representative Western blot of PPARγ and GAPDH (loading control) in C, AA, and SSc monocytes. (top) Densitometric quantification. Each symbol represents the value in arbitrary units normalized against GAPDH from one person. **(B)** (bottom) Representative Western blot showing effect of CSD on PPARγ levels and (top) densitometric quantification (average ± s.e.m.) normalized against GAPDH combining data from three independent experiments using cells from different subjects. The PPARγ level in C monocytes not treated with CSD was set to 100 arbitrary units. **(C)** Demonstration by ICC of decreased expression of caveolin-1 and PPARγ by AA and SSc monocytes. C, AA, and SSc monocytes were stained green for caveolin-1 (Cav-1) and red for PPARγ. Nuclei were counterstained using DAPI (blue). Representative images are shown that are typical of the results obtained in four independent experiments in each category. ^*^p < 0.05, ^**^p < 0.01.

**Figure 4:**
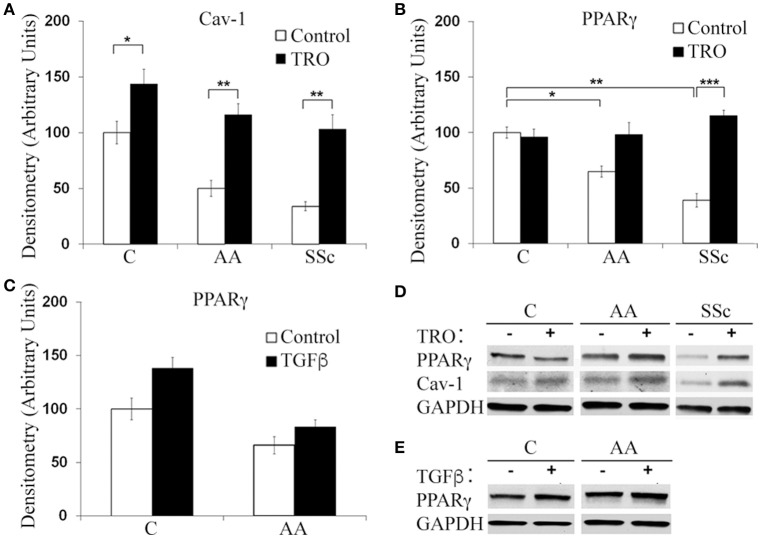
**Effects of TRO and TGFβ on Caveolin-1 and PPARγ Levels in C, AA, and SSc Monocytes**. Isolated C, AA, and SSc monocyes were incubated for 1 h in DMEM/20% FCS (Control) or in medium supplemented with TRO, then extracted and Western blots performed with antibodies against PPARγ, caveolin-1, or GAPDH (loading control). Representative Western blots are shown for Control vs. TRO (called TRO – and +) **(D)** and Control ± TGFβ (called TGFβ – and +) **(E)**. Densitometric quantifications (average ± s.e.m.) normalized against GAPDH combining data from three independent experiments using cells from different subjects are shown in: **(A)** Caveolin-1 quantification from **(D)**. Level in Control C monocytes set to 100 Arbitrary Units. **(B)** PPARγ quantification from **(D)**. Level in Control C monocytes set to 100 Arbitrary Units. **(C)** PPARγ quantification from **(E)**. Level in Control (TGFβ-) monocytes set to 100 Arbitrary Units. ^*^p < 0.05, ^**^p < 0.01, ^***^p < 0.001.

To determine whether a deficiency in PPARγ might contribute to the decreased ability of AA and SSc monocytes to differentiate into ALCs, we examined PPARγ levels in freshly isolated monocytes. Like caveolin-1, PPARγ levels were low in healthy AA monocytes and lower still in SSc monocytes determined both by Western blotting (Figure 3A) and ICC (Figure 3C). PPARγ levels were increased during a 1-h treatment with CSD (Figure 3B), suggesting that caveolin-1 regulates PPARγ accumulation. This conclusion is further supported by long-term ALC differentiation experiments in which CSD increased PPARγ levels in ALCs derived from C monocytes (Figures 1E,F). Conversely, we evaluated the effect of the activation of PPARγ by a short-term treatment with TRO on caveolin-1 and PPARγ levels (Figure 4). While TRO significantly increased both caveolin-1 and PPARγ levels in SSc monocytes, it had an intermediate effect on AA monocytes, and little or no effect on caveolin-1 and PPARγ in C monocytes. Finally, because we have shown that a short-term TGFβ treatment decreases caveolin-1 levels in C monocytes (Tourkina et al., 2010), we evaluated its effect on PPARγ levels. While TGFβ has been reported to decrease PPARγ levels in other cell types (Wei et al., 2010), it slightly increased PPARγ levels in C and AA monocytes (although this effect was not statistically significant) (Figures 4C,D). The combined results suggest that while both caveolin-1 and PPARγ are present at reduced levels in AA and SSc monocytes, these are independent effects.

### Low PPARγ and caveolin-1 levels in healthy AA and SSC patient adipocytes *in vivo*

**Figure 5:**
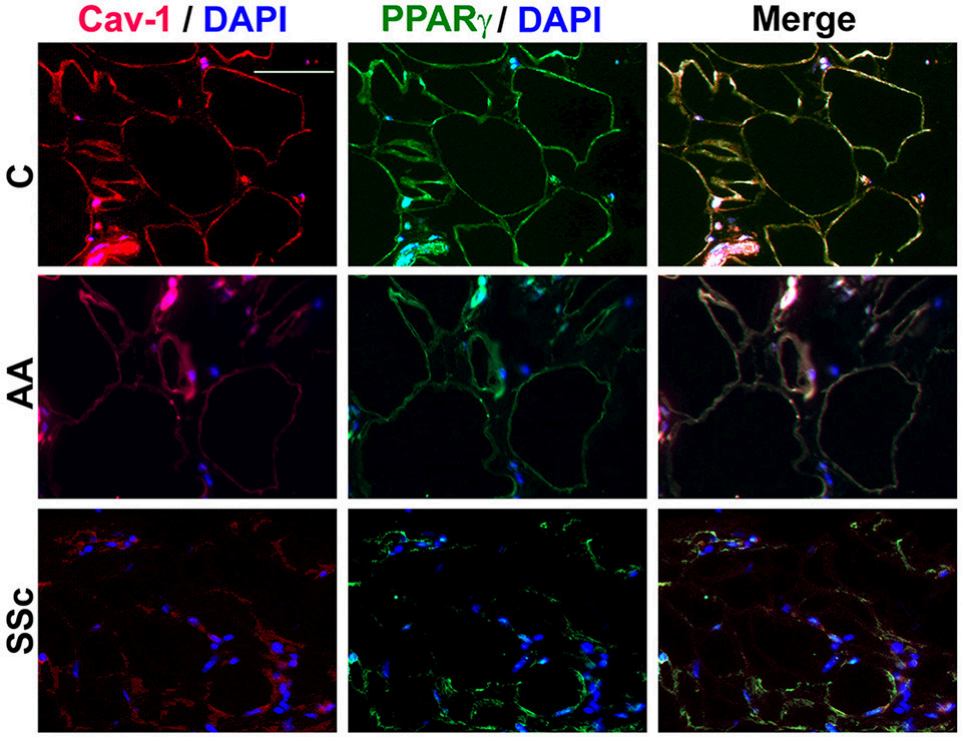
**Low Caveolin-1 and PPARγ Levels in the Subcutaneous Adipose Layer in Healthy AA and SSc Patients**. Caveolin-1 (red) and PPARγ (green) staining are shown at the level of the subcutaneous adipose cell layer. Nuclei were counterstained using DAPI (blue). Similar results were obtained in three independent experiments.

To determine whether the low PPARγ and caveolin-1 expression observed in healthy AA and SSc patient monocytes might also carry over into adipocytes, tissue sections were double-stained for caveolin-1 and PPARγ. Observations at the level of the subcutaneous adipose cell layer demonstrate that while coincident ring staining was observed in healthy C adipocytes, little staining was observed in healthy AA and SSc patient adipocytes (Figure 5). It was also noteworthy that healthy AA and SSc patient adipocytes have a smaller diameter than healthy C adipocytes. Thus, the low caveolin-1/ low PPARγ phenotype of AA and SSc monocytes (that differentiate poorly into ALCs) is shared by the poorly differentiated subcutaneous adipocytes observed in AA and SSc.

### PPARγ and caveolin-1 levels in adipocytes in the mouse model

**Figure 6:**
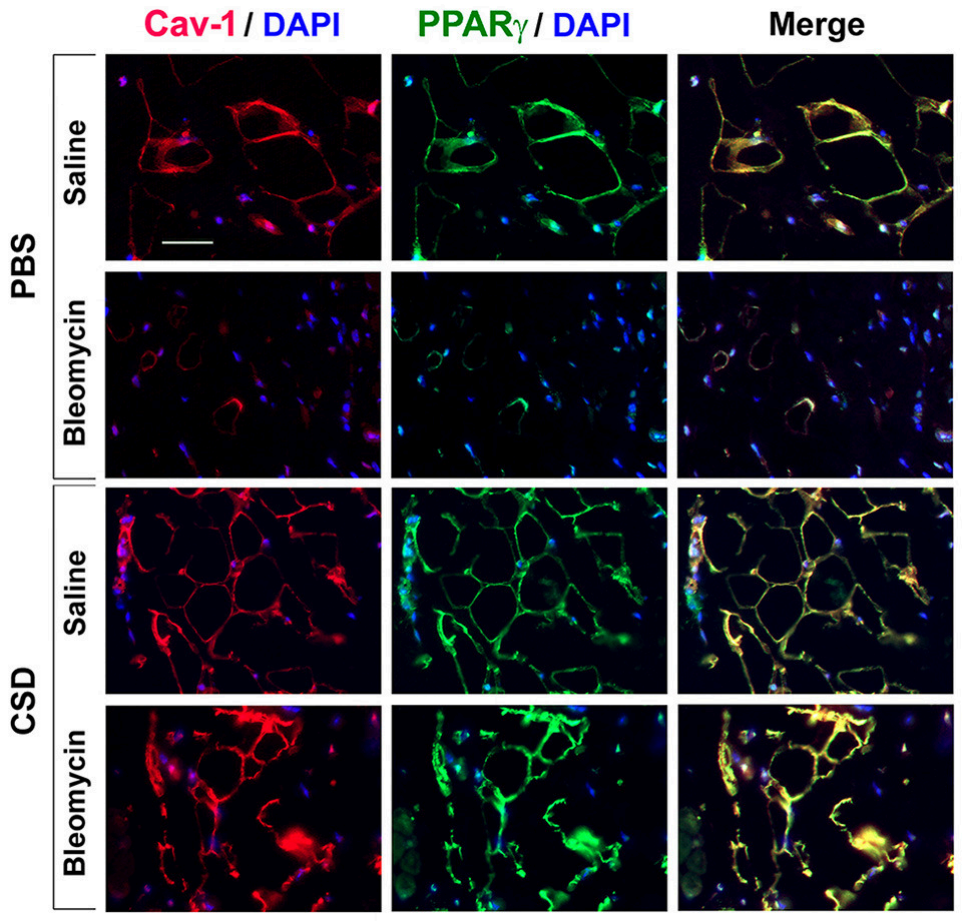
**Low Caveolin-1 and PPARγ Levels in the Subcutaneous Adipose Layer in Fibrotic Mouse Skin**. Mice were treated with Bleomycin or Saline Vehicle and with CSD or PBS Vehicle (Lee et al., 2014a) as indicated. Sections were stained by IHC using anti-Caveolin-1 (red), anti-PPARγ (green), and with DAPI (blue) to detect nuclei. Staining at the level of the subcutaneous adipocyte layer is shown. Similar results were obtained in three independent experiments.

To evaluate the relationship between fibrosis and adipogenesis *in vivo*, we used a mouse model system in which systemic treatment with bleomycin results both in dermal fibrosis and a thinning of the subcutaneous adipose layer (Lee et al., 2014a). Because caveolin-1 and PPARγ expression are low in monocytes with low adipogenic potential (Figure 3), we examined their expression in the mouse model. In control mice, caveolin-1 and PPARγ were coincidently expressed in adipocytes at high levels in a peripheral ring (Figure 6) similar to what we observed in human C adipocytes (Figure 5). When mice are treated with bleomycin, caveolin-1 and PPARγ (Figure 6) are strikingly decreased in the few, small adipocytes that remain (similar to SSc patients, Figure 5). When the mice receive CSD in addition to bleomycin, while the diameter of adipocytes was still somewhat smaller than in control animals, the number of adipocytes is restored to almost the control level and their expression of caveolin-1 and PPARγ is at least as high as in control animals. These observations confirm the importance of caveolin-1 and PPARγ in the balance between fibrosis and adipogenesis both *in vitro* and *in vivo*.

### Fibrosis and loss of subcutaneous adipocytes in a mouse model system

**Figure 7:**
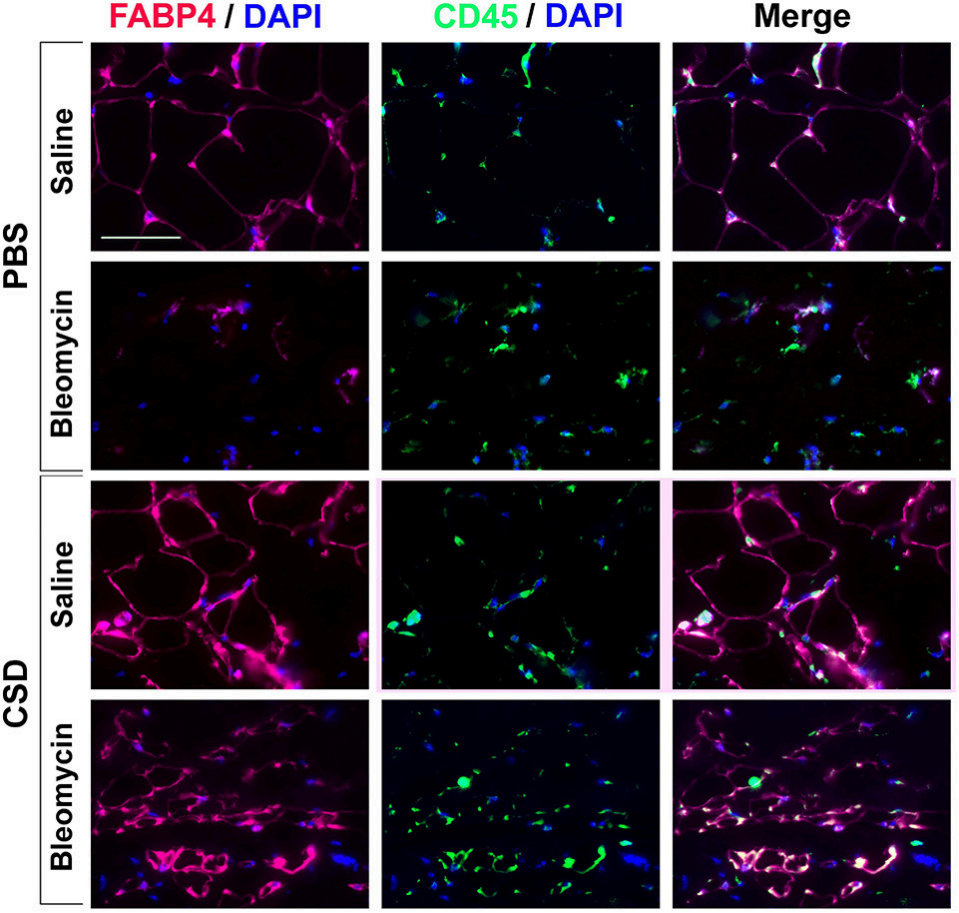
**FABP4 Staining in the Subcutaneous Adipose Layer**. Mice were treated with Bleomycin or Saline Vehicle and with CSD or PBS Vehicle (Lee et al., 2014a) as indicated. Sections from these mice were stained by IHC using anti-FABP4 (red), CD45 (green) and with DAPI (blue) to detect nuclei. Staining at the level of the subcutaneous adipocyte layer is shown. Similar results were obtained in three independent experiments.

To determine whether hematopoietic cells are precursors of adipocytes, skin sections were double-labeled with FABP4 and CD45 (Figure 7). Control mice (no bleomycin/no CSD) showed punctate staining for CD45 and fine ring staining of adipocytes for FABP4. When mice were treated with CSD alone, FABP4 staining was enhanced while CD45 staining remained punctate. When mice were treated with bleomycin alone, in accord with the loss of the adipose layer observed histochemically (Lee et al., 2014a), there was almost no FABP4 staining while CD45 staining was again punctate. Finally, when mice received both bleomycin and CSD, FABP4 staining was enhanced and in some cells FABP4 ring staining coincided with CD45 ring staining, although punctate CD45 staining was also observed. While it is likely that punctate CD45 staining represents macrophages, cells with coincident ring staining with FABP4 and CD45 may be BM-derived adipocytes that contribute to the repair of the injured adipose layer (Bleomycin + CSD), but do not appear in the uninjured adipose layer.

**Figure 8:**
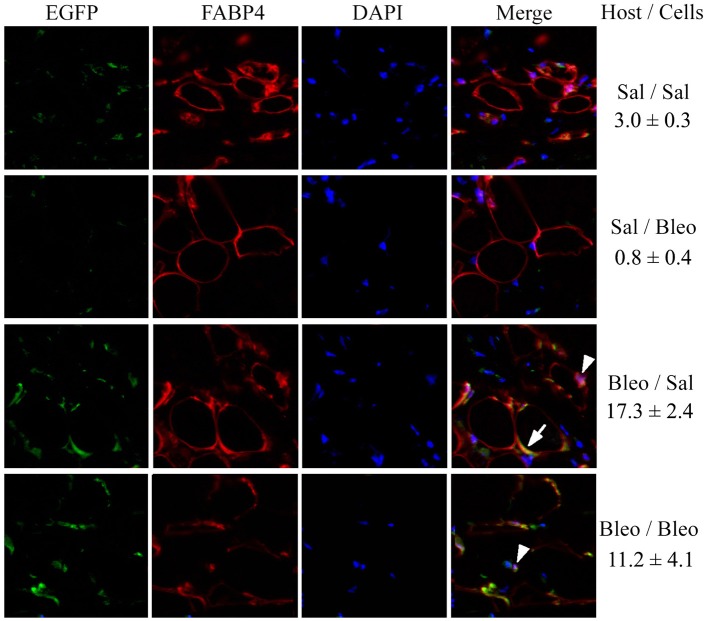
**EGFP+/FABP4+ Cells in the Subcutaneous Adipose Layer**. As described in the Methods, recipient mice, and donor mice were treated with bleomycin or saline vehicle. Monocytes were isolated from the BM of donor mice and injected into the circulation of recipient mice. After sacrifice, EGFP+ cells, FABP4+ cells, and DAPI+ nuclei in skin tissue sections were imaged by fluorescent microscopy for each of the indicated combinations of host mice and donor cells. The number of cells per field that contained EGFP+/FABP4+ ring staining of the adipocyte plasma membrane is indicated (three mice per condition, six fields per mouse).

To confirm the contribution of hematopoietic cells to regeneration of the subcutaneous adipose layer, we traced the fate of EGFP+ monocytes introduced into the circulation of bleomycin-treated and vehicle-treated mice. These “green” cells (which could only have been derived from the injected monocytes) became FABP4+ and incorporated into the adipocyte layer of bleomycin-treated mice much more extensively than they incorporated into the adipocyte layer of vehicle-treated mice (Figure 8) (*p* < 0.01). While this regenerative effect was observed both with injected EGFP+ monocytes derived from vehicle-treated and bleomycin-treated mice, this beneficial effect was somewhat greater with vehicle-treated monocytes (Figure 8). Moreover, the coincident EGFP and FABP4 staining was more ring-like when the injected cells were from control mice and was more punctate when the injected cells were from bleomycin-treated mice. It has been suggested that ring staining of adipocytes with BM markers is due to a crown of macrophages surrounding necrotic adipocytes (Cinti et al., 2005; Berry and Rodeheffer, 2013). This viewpoint was supported by showing multiple nuclei in the ring (Berry and Rodeheffer, 2013). While we observe some punctate staining that represents macrophages in Figure 8 (examples are marked with arrowheads), we also detect coincident EGFP/FABP4 ring staining (an example is marked with an arrow) without multiple nuclei, suggesting that the coincident ring staining is due to BM-derived cells that express high levels of FABP4, not BM-derived macrophages that surround FABP4+ cells. Thus, while there are BM-derived macrophages in subcutaneous adipose tissue in all cases, we propose that repair of bleomycin-induced injury occurs due to the differentiation of healthy monocytes into ALC.

**Figure 9:**
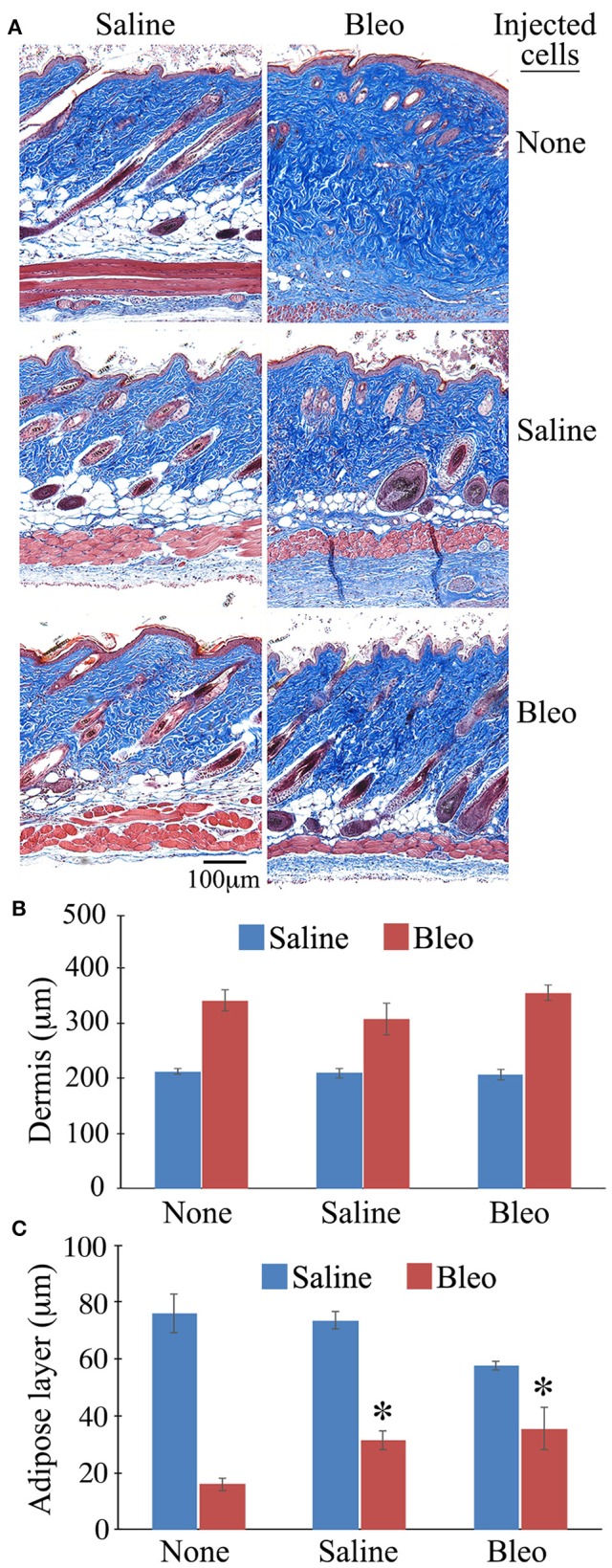
**Thickness of the Dermis and the Subcutaneous Adipose Layer in Mice Injected with BM-Derived Monocytes**. Skin tissue sections from the mice described in Figure 8 as well as host mice treated with bleomycin or saline and receiving no cell injection were stained using Massons' Trichrome Stain and the thickness of the dermis and the subcutaneous adipose layer measured (three mice per category, six sites per mouse). **(A)** Representative images of Massons' staining, **(B)** Quantification of thickness of dermis, **(C)** Quantification of thickness of subcutaneous adipose layer. ^*^p < 0.05 compared to bleomycin-treated mice receiving no injection.

When the same sections were stained with Masson's Trichrome, as expected, bleomycin treatment reduced the thickness of the subcutaneous adipose layer and this effect was reversed in mice injected with either healthy monocytes or monocytes from bleomycin-treated mice (Figure 9). While the data shown in Figure 9 suggest that monocyte injection decreases the thickness of the dermis, the effect was not statistically significant.

## Discussion

Here we report several novel observations regarding monocytes and altered adipogenesis in fibrotic disease including: (1) SSc monocytes and healthy AA monocytes have a decreased ability to differentiate into adipocytes *in vitro* compared to healthy C monocytes both in the absence and the presence of TRO (a synthetic PPARγ ligand that induces adipocyte differentiation); (2) Healthy AA and SSc monocytes and healthy AA and SSc adipocytes (*in vivo*) are deficient in both caveolin-1 and PPARγ; (3) CSD enhances both ALC differentiation by C, AA, and SSc monocytes *in vitro* and PPARγ expression; (4) When mice are treated systemically with bleomycin, dermal fibrosis occurs, adipocytes lose caveolin-1 and PPARγ (as in SSc patients and healthy AA), and the subcutaneous adipose layer shrinks; (5) CSD treatment of bleomycin-treated mice promotes subcutaneous adipogenesis and the expression of PPARγ in adipocytes (just as it promotes PPARγ expression in SSc and AA monocytes and their differentiation into ALCs); (6) Many of the cells involved in the repair of the bleomycin-injured adipose layer are BM-derived monocytes as evidenced by their expression of CD45 and by the contribution of injected EGFP+ monocytes to the adipose layer.

Our studies and the literature indicate several connections between caveolin-1 and PPARγ that may be relevant to the balance between fibrosis and adipogenesis. Both caveolin-1 and PPARγ are present at low levels in monocytes from SSc patients and healthy AA that exhibit an enhanced ability to differentiate into fibrocytes/myofibroblasts (Reese et al., 2014b) and an inhibited ability to differentiate into adipocytes (Figure 1). SSc and AA subcutaneous adipocytes (Figure 5) and SSc fibroblasts are also deficient in caveolin-1 and PPARγ (Tourkina et al., 2005; Del Galdo et al., 2008; Wei et al., 2010). Both caveolin-1 and PPARγ are among the group of proteins in which mutation results in lipodystrophy (Rochford, 2010). Both are downregulated in cultured adipocytes during bacterial infection (Nagajyothi et al., 2008) and in a variety of cell types by TGFβ treatment (Wang et al., 2006; Tourkina et al., 2010; Wei et al., 2010). However, data from our lab and others suggests that TGFβ does not decrease PPARγ levels in monocytes (Figure 4, Kintscher et al., 2002). Caveolin-1 and PPARγ can also regulate each other's expression and/or activation. CSD (which acts as a surrogate for caveolin-1) increases PPARγ levels in C, AA, and SSc monocytes (Figure 4). Caveolin-1 can activate PPARγ in HEK293 cells (Burgermeister et al., 2011). Overexpression of PPARγ or its activation by rosiglitazone or TRO can increase caveolin-1 expression in THP-1 cells (Llaverias et al., 2004) and peripheral blood monocytes (especially AA and SSc monocytes, Figure 4).

We previously observed that the differentiation *in vitro* of monocytes into fibrocytes/myofibroblasts is enhanced in cells from healthy AA and SSc patients compared to healthy C and must be due to the relative lack of caveolin-1 in these cells in that it is inhibited by CSD (Reese et al., 2014b). Here we report that, conversely, the differentiation *in vitro* of monocytes into ALCs is inhibited in cells from healthy AA and SSc patients compared to healthy C. This difference is observed both in the presence or absence of TRO (which promotes ALC differentiation). Moreover, just as CSD inhibits the enhanced fibrogenic differentiation of AA and SSc monocytes, it enhances their adipogenic differentiation suggesting that caveolin-1 both inhibits fibrosis and promotes adipogenesis. The same molecular mechanisms are likely to be enhancing the fibrogenic differentiation and inhibiting the adipogenic differentiation of AA and SSc monocytes, given that both AA and SSc monocytes are deficient in both caveolin-1 and PPARγ. Besides their direct effects on monocyte differentiation, the ability of caveolin-1 and PPARγ to work together to regulate monocyte differentiation may be enhanced because experiments with CSD and TRO indicate that each of these proteins positively regulates the expression of the other in monocytes.

Because macrophages can express FABP4 (Jiang et al., 2013; Lázaro et al., 2013), expression of FABP4 is not a sufficient criterion to identify cells as adipocytes. Therefore, we determined the phenotype of our AIM-induced ALCs in comparison macrophages polarized using either M-CSF or GM-CSF (Figure 2). While we confirmed that macrophages express FABP4, the level of expression of FABP4 was much higher in ALCs than in macrophages. Moreover, macrophages were positive for CD14, CD163, CD206, Arginase, and iNOS while ALCs were negative. Our data are in agreement with the work of Kuwana's group on the differentiation of CD14+ monocytes into adipocytes accompanied by the loss of CD14 (Kuwana et al., 2003). Thus, it is not necessary to isolate CD14- monocyte-derived fibrocytes (Hong et al., 2005) in order to differentiate monocyte-derived cells into adipocytes.

Just as CSD partially reverses the inhibition of adipogenic differentiation of AA and SSc monocytes *in vitro* (Figure 1), it has analogous effects *in vivo* on mice treated systemically with bleomycin. We previously showed that, in addition to thickening of the dermis, mice treated systemically with bleomycin lose subcutaneous fat and that these effects are blocked by CSD (Lee et al., 2014a). Monocytes and monocyte-derived fibrocytes have been shown to be precursors for several cell types including fibrocytes and adipocytes (Kuwana et al., 2003; Hong et al., 2005, 2007). Here we have extended our previous studies to show that, in mouse subcutaneous adipose tissue treated with bleomycin, almost no FABP4+ cells are observed. However, when mice receive bleomycin followed by CSD, a high level of FABP4 ring staining of adipocytes is observed that appears to coincide with CD45 ring staining in some cells (Figure 7). It has been proposed that under pathological conditions (e.g., obesity), coincident CD45/FABP4 ring staining occurs that is due to a crown of macrophages surrounding necrotic adipocytes (Cinti et al., 2005; Berry and Rodeheffer, 2013). While we observed individual macrophages in Figure 7 and we observed coincident CD45/FABP4 ring staining, we did not observe multiple nuclei in the rings, suggesting that they are not crowns but are double-positive adipocytes.

To further evaluate the possibility that BM-derived cells contribute to the repair of injury to the subcutaneous adipose layer, we injected EGFP+ BM into control mice and mice treated with bleomycin. Injection of EGFP+ BM has previously allowed the demonstration that these cells can differentiate into adipocytes *in vivo* in mice receiving a high-fat diet or treated with rosiglitazone (Crossno et al., 2006). However, these authors drew the conclusion that their EGFP+ adipocytes were derived from mesenchymal stromal cells (and not macrophages) because their EGFP+ adipocytes were CD45−/CD11b−. When we used this approach (Figure 8), we also observed that the injected cells appear to differentiate into FABP4+ adipocytes, not a multinucleate crown of macrophages. We conclude that our results further validate the contribution of monocytes to the recovery of the subcutaneous adipose layer from bleomycin-induced injury.

In agreement with our observations in Figure 7, when we injected EGFP+ monocytes we observed a major contribution of these cells to the adipose layer in bleomycin-treated mice but not in control mice. There are several reasonable potential explanations for the discrepancy between our results and those of Crossno et al. (2006) (1) The models are different (bleomycin treatment vs. high-fat diet or treatment with rosiglitazone), (2) The adipose tissues being studied are different (subcutaneous vs. omental or dorsal intracapsular brown fat), (3) Crossno et al. (2006) isolated adipocytes by flotation. The adipocytes in the subcutaneous fat of bleomycin-treated mice injected with monocytes are relatively small (Figure 9A) and may not float, (4) Crossno et al. (2006) appears to have performed flow cytometry using live cells. We find that certain cell types need to be fixed and permeabilized to reveal CD45 staining (not shown). (5) It is possible that Crossno et al. (2006) do not detect CD45 on their EGFP+ adipocytes because the progenitors may have lost CD45 during adipocyte differentiation as has been observed during the differentiation of monocytes into adipocytes *in vitro* (Kuwana et al., 2003). In any case, we are confident that the cells that we injected that differentiate into adipocytes are monocytes and not mesenchymal stromal cells because they have the proper phenotype (CD45+/CD11b+/CD68+/CD73−/CD90−/CD105−).

We have also extended our previous studies to examining the levels of caveolin-1 and PPARγ in subcutaneous adipose tissue. Coincident ring staining is observed in control tissue. Both caveolin-1 and PPARγ are almost totally lost due to bleomycin treatment; however, CSD treatment after bleomycin restores the coincident ring staining of caveolin-1 and PPARγ. The combined observations suggest that adipocytes are derived to a large extent from hematopoietic cells, that skin fibrosis may result from an altered balance in the differentiation of monocytes into fibrocytes/myofibroblasts and adipocytes, and that caveolin-1 and PPARγ are key players in regulating this balance.

Interestingly, the injection of BM-derived monocytes also resulted in a significant recovery in the thickness of the subcutaneous adipose layer (Figure 9). Besides using bleomycin-treated and vehicle-treated mice as recipients of EGFP+ cells, we also compared the function of EGFP+ cells from bleomycin-treated and vehicle-treated mice as the donor cells. EGFP+ cells from vehicle-treated mice were marginally more effective in incorporating into the adipose layer. The importance of these differences between EGFP+ cells from bleomycin- and vehicle-treated mice will require further study. It may be that when the bleomycin BM monocytes are taken out of the fibrotic environment and cultured overnight, they lose their pro-fibrotic phenotype in this assay. This could be analogous to the situation that occurs when scleroderma patients are injected with autologous adipose-derived mesenchymal stem cells and beneficial effects are observed, despite the fact that these cells are derived from an individual (the patient himself) with fibrotic disease (Christopeit et al., 2008; Guiducci et al., 2010; Keyszer et al., 2011; Scuderi et al., 2013). Finally, it should be noted that, while few in number, there are previous publications indicating a beneficial effect of monocyte injection (e.g., injection of monocytes resulted in clearance of plaques in a model of Alzheimer's disease, Hohsfield and Humpel, 2015).

In summary, the current study strongly supports and extends our observations on the role of monocytes and cells derived from monocytes (e.g., fibrocytes/myofibroblasts and adipocytes) in skin fibrosis and on the predisposition of AA to fibrotic diseases. Our findings highlight the idea that signaling molecules that regulate monocyte differentiation (e.g., caveolin-1, PPARγ) are promising targets for novel treatments for fibrotic diseases such as SSc. In particular, CSD reverses both the enhanced fibrogenic differentiation of monocytes and their inhibited adipogenic differentiation both *in vitro* and *in vivo*. The physiological relevance of the results of this study is strongly supported by the similarity in the observations we have made in human and mouse tissues. We observed a thinning of the subcutaneous adipose cell layer in healthy AA, SSc patients, and in mice treated systemically with bleomycin. Moreover, in all these cases the remaining adipocytes were deficient in both caveolin-1 and PPARγ. Together these observations strongly suggest that caveolin-1 and PPARγ work together to maintain the proper balance between the differentiation of monocytes into myofibroblasts and adipocytes, and that fibrotic disease results when this balance is upset to favor myofibroblast differentiation.

## Ethics statement

This study was carried out in accordance with the recommendations of the Medical University of South Carolina Institutional Review Board for Human Research with written informed consent from all subjects. All subjects gave written informed consent in accordance with the Declaration of Helsinki. The protocol was approved by the Medical University of South Carolina Institutional Review Board for Human Research. This study was carried out in accordance with the recommendations of the Medical University of South Carolina Institutional Animal Care and Use Committee. The protocol was approved by the Medical University of South Carolina Institutional Animal Care and Use Committee.

## Author contributions

RL and CR participated in study design, performing experiments, data interpretation, and manuscript preparation. GC, BP, MZ, and CW participated in performing experiments. MB participated in performing experiments and in editing the manuscript. RS participated in editing the manuscript. SH participated in study design, data interpretation, and editing the manuscript. KH participated in data interpretation. ET participated in study design, data interpretation, manuscript preparation, and editing the manuscript. All authors read and approved the final manuscript.

## Funding

This work was supported by grants: NIH NIAMS R01 AR062078 and a grant from the Scleroderma Foundation (to ET); USARMY/USAMRAA W81XWH-11-1-0508 (to SH); NIH NIAMS P60 AR049459 (Multidisciplinary Clinical Research Center) (to RS); and an NIH NCRR Construction Grant C06 RR015455. ET also received the Marta Max Award from the Scleroderma Foundation.

### Conflict of interest statement

The authors declare that the research was conducted in the absence of any commercial or financial relationships that could be construed as a potential conflict of interest.
